# Fluoroquinolones and the risk of aortic aneurysm or dissection: A population‐based propensity score‐matched German cohort study

**DOI:** 10.1002/phar.70020

**Published:** 2025-04-26

**Authors:** Julia Wicherski, Jonas Peltner, Cornelia Becker, Katrin Schüssel, Gabriela Brückner, Andreas Schlotmann, Helmut Schröder, Winfried V. Kern, Britta Haenisch

**Affiliations:** ^1^ Federal Institute for Drugs and Medical Devices (BfArM) Research Division, Pharmacoepidemiology Bonn Germany; ^2^ German Centre for Neurodegenerative Diseases (DZNE) Pharmacoepidemiology in Neurodegenerative Disorders Bonn Germany; ^3^ Research Institute of AOK (WIdO) Berlin Germany; ^4^ Division of Infectious Diseases, Department of Medicine II University of Freiburg Faculty of Medicine and Medical Centre Freiburg Germany; ^5^ Centre for Translational Medicine University of Bonn Bonn Germany

**Keywords:** adverse drug reactions, aortic aneurysm, fluoroquinolones, macrolides

## Abstract

**Objective:**

To investigate the risk of aortic aneurysm or dissection associated with fluoroquinolone (FQ) prescription compared to macrolides in German routine health care data in order to replicate the recent study (*Pharmacotherapy* 2023;43:883) extending the results by contributing evidence for six additional broad‐spectrum antibiotic classes as active comparators.

**Design:**

Cohort study in active comparator new user design comparing FQ with macrolides, tetracyclines, penicillins with extended spectrum, penicillins and beta‐lactamase inhibitor combinations, second‐ and third‐generation cephalosporins, sulfonamide and trimethoprim combinations, and lincosamides.

**Setting:**

German statutory health insurance, the “Allgemeine Ortskrankenkasse” (AOK), January 2013 to December 2019.

**Participants:**

Adults with at least one new prescription fill for FQ or active comparator antibiotics. New users were defined as individuals without antibiotic prescription fills, aortic aneurysm or dissection diagnoses, and hospitalization within 365 days prior to the cohort entry date. Users of FQ and active comparators were matched by nearest neighbor 1:1 propensity score matching.

**Main Outcome Measures:**

Incident inpatient aortic aneurysm or dissection was observed within a 60‐day risk window. In sensitivity analyses, an extended risk window of 90 days was applied, and specific FQ agents, dosages, and diagnoses were stratified.

**Results:**

FQ episodes were associated with an increased risk for aortic aneurysm or dissection compared to macrolides (aHR = 1.52 [1.33; 1.74]), which replicates the risk estimate of Garg et al. (aHR = 1.34 [1.17; 1.54]). This association was robust in a 90‐day risk window and for ciprofloxacin, levofloxacin, and moxifloxacin. Moxifloxacin comprised the greatest risk of aortic aneurysm or dissection compared to macrolides (aHR = 2.13 [1.64; 2.77]). Moreover, we observed similar associations when comparing FQ to tetracyclines, penicillins with extended spectrum, cephalosporins, and lincosamides (aHR = 1.86 [1.54; 2.24], aHR = 1.45 [1.28; 1.65], aHR = 1.23 [1.10; 1.37], and aHR = 1.73 [1.43; 2.11]), respectively.

**Conclusion:**

In a German cohort study, FQ use was associated with a 52% increased risk for aortic aneurysm or dissection within 60 days compared with macrolide use. The risk of FQ‐associated aortic aneurysm or dissection compared to macrolides can be replicated in German routine health care data. Extending the analysis, we provided new insights that the effect size may depend on the chosen AC.

## 1 INTRODUCTION

Fluoroquinolones (FQ) are considered reserve broad‐spectrum antibiotics with high efficacy against gram‐positive, gram‐negative, atypical, and anaerobic bacteria.^1^ However, increasing evidence for FQ‐associated aortic aneurysm and dissection and further serious adverse drug reactions led to changes in authorization in 2019 by the European Medicines Agency (EMA).^2^ Nevertheless, FQs are still prescribed frequently.^3^

Potential mechanisms underlying FQ‐associated adverse drug reactions are oxidative stress triggered by FQ,^4^ and the degrading effect of FQ on collagen, introducing an increased activity of matrix metalloproteinase with negative consequences for the structure of the extracellular matrix.^5^ Both mechanisms can damage the aortic wall, leading to a higher risk of aortic aneurysm and dissection.

The evidence for FQ‐associated aortic aneurysm and dissection was pointed out in several pharmacoepidemiologic studies, especially since the 2019 EMA risk assessment report, but results were as heterogeneous as their designs,^6^, ^7^, ^8^, ^9^, ^10^, ^11^, ^12^, ^13^, ^14^ and evidence from large European countries is scarce. At the time of writing, the most recent study^6^ evaluating FQ‐associated aortic aneurysm or dissection was conducted using United States claims data from MarketScan and Medicare and compared new FQ with new macrolide prescription fills, but the US study did not compare FQ to other antibiotics prescribed for similar infections as FQ. The propensity score‐matched US cohort estimated a 34% [95% CI: 17%; 54%] increased relative risk of aortic aneurysm or dissection for FQ initiators compared to macrolide initiators during 60‐day follow‐up.^6^

In this study, we replicated the design of Garg et al.^6^ within a pre‐existing data set from another FQ‐related population‐based cohort study to examine the risk of FQ‐associated aortic aneurysm and dissection in routine care, representing a large European country. Extending the analyses of Garg et al.,^6^ we analyzed not only macrolides but also additional antibiotics in order to assess the differences in risk estimation due to the choice of the reference group.

## 2 METHODS

### 2.1 Source of data

We used longitudinal routine data from one of the largest associations with 11 statutory health insurances in Germany, the “AOK – Die Gesundheitskasse,” which covers about 28 million individuals.^15^ The data set provided for this cohort study comprised the insurance time from January 1, 2013, to December 31, 2019, and contained information on age, gender, outpatient drug prescribing (the German adaptation of the World Health Organization's Anatomical Therapeutic Chemical (ATC) classification with defined daily doses (DDD)^16^) as well as quarterly outpatient and weekly inpatient medical diagnoses (International Classification of Diseases, 10th Edition, German Modification (ICD‐10‐GM)^16^), and inpatient procedure coding (the German adaptation of the International Classification of Procedures in Medicine^16^).

### 2.2 Cohort

**FIGURE 1:**
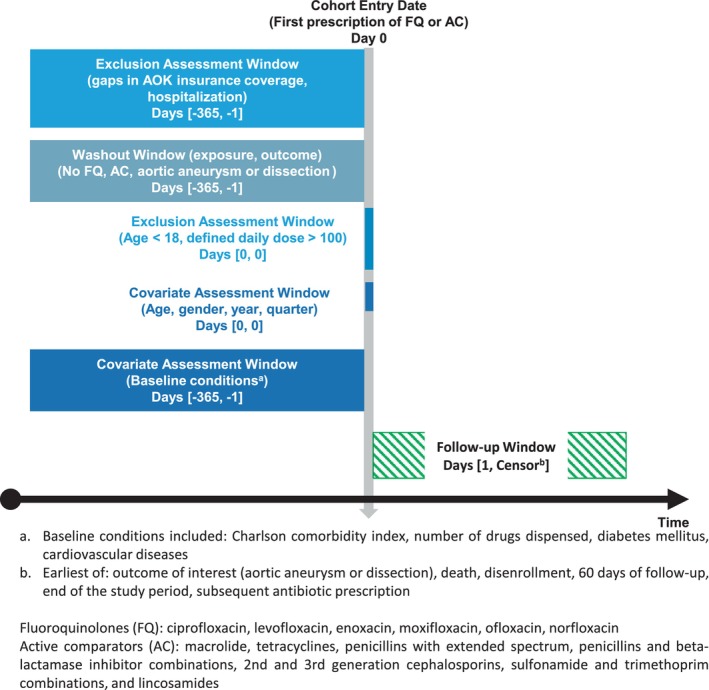
Study design diagram (illustration template from Schneeweiss et al.^17^).

We built our study cohort following the inclusion and exclusion criteria used by Garg et al.^6^ We defined the cohort entry date (CED) as the date of the first prescription fill for FQ or macrolides, tetracyclines, penicillins with extended spectrum, penicillins and beta‐lactamase inhibitor combinations, second‐ and third‐generation cephalosporins, sulfonamide and trimethoprim combinations, and lincosamides. Patients with two or more different antibiotics (ATC codes) dispensed at the index date were excluded. Moreover, individuals with an implausible amount of drug dispensed (either number of drug packages or sum of DDDs above 100) during the study period (years 2013–2019) were excluded. To be included in the study, individuals had to be at least 18 years of age at the CED and continuously insured by the AOK for at least 365 days prior to their CED, which was defined as the date of the first antibiotic prescription after a washout period of 365 days. To be included, individuals were furthermore required to not have had any diagnoses of aortic aneurysm or dissection during the baseline period and not have had a record of any hospitalization or filled prescriptions for more than one study antibiotic on the CED. Since outpatient diagnoses are only captured on a quarterly basis, five quarters (the index quarter and four prior quarters) were used to ensure the 365‐day baseline window for application of exclusion criteria. Figure 1 displays the study diagram of the cohort selection.

Individuals were represented by index episodes, and multiple inclusions into the cohorts were possible with respect to all selection criteria. Individuals were followed up from CED until the earliest occurrence of aortic aneurysm or dissection, end of AOK enrollment, end of the study period or follow‐up, death, or subsequent antibiotic prescription. Minor deviations from Garg et al.^6^ were that we included all routes of administration not only oral ones, and that we were not able to identify the length of application (“at least 3 days of exposure”) because information on drug dosage is not available in German routine health claims data. However, parenteral or intravenous antibiotic prescriptions are very uncommon in the outpatient sector, and dispensing of drugs in Germany is usually by packages covering several days of drug supplies. Moreover, prescription and dispensing of drugs are in general only a proxy for exposure without any control over compliance and actual use of drugs by patients. Another deviation is the length of the washout period for antibiotics, aortic aneurysm or dissection coding, and hospitalizations, which was only 6 months in the original study. Here, we deviated from Garg et al.^6^ because we used a washout period of 12 months.

### 2.3 Aortic aneurysm and dissection

The primary outcome measured was the incidence of aortic aneurysm or dissection requiring hospitalization within a 60‐day risk window. Therefore, we counted only the first occurrence of inpatient discharge diagnoses of I71 codes of the ICD‐10‐GM. For the exclusion criteria during the baseline window, all inpatient discharge diagnoses and outpatient diagnoses of ICD‐10‐GM: I71, which are labeled as “confirmed,” were marked as relevant cases to exclude in order to ensure the observation of new cases only.

### 2.4 Statistical analysis

Standardized differences were estimated to quantify differences in the distribution of baseline characteristics for gender, age, Charlson comorbidity index (CCI),^18^, ^19^ diabetes mellitus, and cardiovascular diseases before and after 1:1 nearest neighbor propensity score matching with a 0.1 caliper. Propensity scores were estimated by logistic regression. Variables included for this approach were age in years, gender, CCI, number of drugs dispensed at baseline, diabetes mellitus (ICD‐10‐GM: E10‐E14), and cardiovascular diseases (ICD‐10‐GM: G45, G46, I11, I13, I20‐I22, I24, I25, I34‐I37, I42‐I50, I61‐I67, I70, I72‐I74, I77, J81, K55).

Incidence rates of aortic aneurysm or dissection were reported as the number of aortic aneurysm or dissection cases per 10,000 person‐years. Incidence rates were standardized by the German standard population, which is based on the Micro Census 2011.^20^ Adjusted hazard ratios (aHR) were estimated by applying Cox proportional hazard regression models with additional covariate adjustment for age, gender, diabetes mellitus, cardiovascular diseases, and CCI.

We performed subgroup analyses for age (≤50‐year‐olds, >50‐year‐olds), gender (males, females), and with or without diabetes mellitus or cardiovascular diseases to disaggregate our effect estimates for important patient characteristics. Moreover, we conducted sensitivity analyses for the outcome itself by stratification into aortic aneurysm or dissection only as well as single‐agent comparisons of FQ agents and prolonged the risk window from 60 to 90 days of follow‐up. Instead of stratification for exposure duration, we alternatively used DDDs as a proxy and disaggregated our analysis into low‐, medium‐, and high‐dose categories to check for dose–response effects. The dose categories were based on the distribution of the DDDs dispensed per index prescription of the respective active ingredient in the cohort. If an individual's DDD amount was in the lower third (i.e., lower than the 33rd percentile) of all dispensings, the case was classified as low dosage. DDD amounts between the 33rd and 66th percentiles were defined as medium dosage, and DDD amounts above the 66th percentile were defined as high dosage. Due to a very small number of individuals with active comparator episodes with low dosage after matching, this category was combined with the medium‐dose category. Lastly, we used a random effects (frailty) model in a sensitivity analysis in order to account for unobserved heterogeneity.

The propensity score matching was conducted using SAS software, version 9.4 (SAS Institute, Inc., *North Carolina, USA*). All other analyses were performed in R, version 4.1.0, from January 2024 to December 2024. The analyzed data set was based on a sample size calculation able to report hazard ratios ≥1.20 with a power of 80%. All effect estimates were reported with their corresponding 95% confidence intervals (CI).

## 3 RESULTS

**FIGURE 2:**
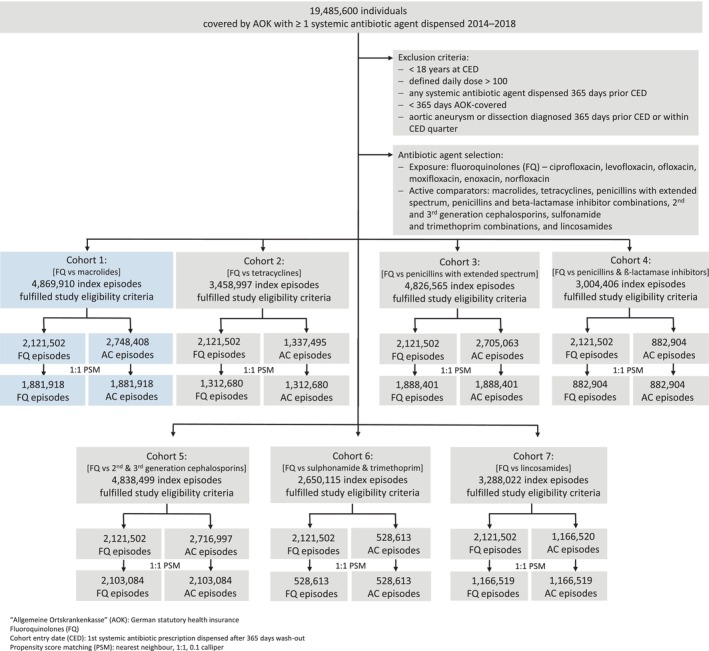
Cohort attrition.

We identified 2,121,502 FQ episodes, which fulfilled all selection criteria. Among the selected active comparators, 2,748,408 macrolide episodes were selected. After the propensity score matching approach, the analysis was based on 1,881,918 index episodes in each group. Figure 2 illustrates the cohort attrition for all seven separate data sets. The replication of the FQ and macrolide comparison of Garg et al.^6^ (i.e., cohort 1) is highlighted in blue.

Before the propensity score matching, individuals with FQ episodes had a higher mean age (standardized mean difference 0.490). Patients in FQ episodes were on average 57 years old, whereas patients in macrolide episodes were on average 47 years old. Moreover, patients during FQ episodes had a higher comorbidity burden as measured by CCI, drugs dispensed, and diagnoses of diabetes mellitus or cardiovascular diseases. After propensity score matching, all baseline conditions of FQ episodes compared to active comparator episodes were well‐balanced (see Table 1). Compared to the other active comparators, individuals with FQ episodes were again on average older than individuals with episodes of tetracyclines, penicillins with extended spectrum, second‐ and third‐generation cephalosporins, and lincosamides before the propensity score matching (standardized mean difference >0.2). Individuals with episodes of penicillins with beta‐lactamase inhibitors were more frequently male, whereas individuals with episodes of sulfonamides and trimethoprim combinations were predominantly female (standardized mean difference >0.2, see Tables S1a–f).

**TABLE 1:** Study population characteristics before and after propensity score matching, cohort FQ vs. macrolides.

	Before propensity score matching		Standardized difference	After propensity score matching		Standardized difference
	FQ (*n* = 2,121,502)	MAC (*n* = 2,748,408)		FQ (*n* = 1,881,918)	MAC (*n* = 1,881,918)	
Age, years (mean (SD))	56.51 (19.32)	47.32 (18.18)	0.490	53.87 (18.80)	53.03 (17.98)	0.046
Male gender (%)	868,490 (40.94)	1,218,568 (44.34)	0.069	815,683 (43.34)	752,997 (40.01)	0.068
CCI (%)						
0	1,295,906 (61.08)	1,916,428 (69.73)	0.213	1,193,354 (63.41)	1,226,679 (65.18)	0.046
1–2	615,584 (29.02)	683,089 (24.85)		528,927 (28.11)	515,870 (27.41)	
3–4	162,410 (7.66)	120,988 (4.40)		128,705 (6.84)	112,168 (5.96)	
5+	47,602 (2.24)	27,903 (1.02)		30,932 (1.64)	27,201 (1.45)	
Drugs dispensed (%)						
0	330,966 (15.60)	621,737 (22.62)	0.360	330,580 (17.57)	329,016 (17.48)	0.031
1–3	436,333 (20.57)	759,734 (27.64)		432,857 (23.00)	433,353 (23.03)	
4–10	518,373 (24.43)	695,722 (25.31)		475,612 (25.27)	496,004 (26.36)	
11–20	382,195 (18.02)	364,333 (13.26)		324,689 (17.25)	322,732 (17.15)	
21+	453,635 (21.38)	306,882 (11.17)		318,180 (16.91)	300,813 (15.98)	
Diabetes mellitus (%)						
E10‐14	410,669 (19.36)	312,530 (11.37)	0.223	299,090 (15.89)	294,204 (15.63)	0.007
Cerebral vascular syndromes (%)						
G45	19,816 (0.93)	14,298 (0.52)	0.049	13,952 (0.74)	13,647 (0.73)	0.002
G46	1345 (0.06)	854 (0.03)	0.015	958 (0.05)	823 (0.04)	0.003
Hypertension (%)						
I11	117,528 (5.54)	86,603 (3.15)	0.117	83,333 (4.43)	82,979 (4.41)	0.001
I13	4425 (0.21)	2637 (0.10)	0.029	2873 (0.15)	2568 (0.14)	0.004
Ischemic heart disease (%)						
I20	38,748 (1.83)	31,290 (1.14)	0.057	28,638 (1.52)	28,975 (1.54)	0.001
I22	472 (0.02)	353 (0.01)	0.007	376 (0.02)	340 (0.02)	0.001
I24	4879 (0.23)	3441 (0.13)	0.025	3476 (0.19)	3333 (0.18)	0.002
I25	247,471 (11.67)	166,286 (6.05)	0.199	173,824 (9.24)	162,474 (8.63)	0.021
Valve diseases (%)						
I34	70,978 (3.35)	55,142 (2.01)	0.083	49,981 (2.66)	51,226 (2.72)	0.004
I35	57,298 (2.70)	40,332 (1.47)	0.086	39,238 (2.09)	38,738 (2.06)	0.002
I36	7498 (0.35)	5706 (0.21)	0.028	5116 (0.27)	5337 (0.28)	0.002
I37	2440 (0.12)	2316 (0.08)	0.010	1800 (0.10)	1902 (0.10)	0.002
Cardiomyopathy (%)						
I42	18,740 (0.88)	13,860 (0.50)	0.046	14,039 (0.75)	12,964 (0.69)	0.007
I43	959 (0.05)	842 (0.03)	0.007	701 (0.04)	784 (0.04)	0.002
Cardiac arrhythmia (%)						
I44	33,728 (1.59)	23,027 (0.84)	0.069	23,625 (1.26)	22,017 (1.17)	0.008
I45	21,861 (1.03)	18,962 (0.69)	0.037	16,845 (0.90)	16,397 (0.87)	0.003
I46	481 (0.02)	360 (0.01)	0.007	355 (0.02)	333 (0.02)	0.001
I47	24,512 (1.16)	24,081 (0.88)	0.028	19,338 (1.03)	20,368 (1.08)	0.005
I48	105,150 (4.96)	60,933 (2.22)	0.148	69,465 (3.69)	59,858 (3.18)	0.028
I49	129,689 (6.11)	107,270 (3.90)	0.101	96,275 (5.12)	96,077 (5.11)	<0.001
Heart failure (%)						
I50	152,715 (7.20)	96,001 (3.49)	0.165	103,039 (5.48)	93,912 (4.99)	0.022
Cerebrovascular diseases (%)						
I61	3139 (0.15)	1967 (0.07)	0.023	2221 (0.12)	1815 (0.10)	0.007
I62	1034 (0.05)	680 (0.03)	0.013	725 (0.04)	632 (0.03)	0.003
I63	21,214 (1.00)	12,896 (0.47)	0.062	14,830 (0.79)	12,465 (0.66)	0.015
I64	20,752 (0.98)	12,028 (0.44)	0.065	14,238 (0.76)	11,683 (0.62)	0.016
I65	54,031 (2.55)	37,660 (1.37)	0.085	37,909 (2.01)	36,793 (1.96)	0.004
I66	2550 (0.12)	1983 (0.07)	0.016	1838 (0.10)	1827 (0.10)	<0.001
I67	71,670 (3.38)	47,481 (1.73)	0.105	49,596 (2.64)	46,293 (2.46)	0.011
I68	215 (0.03)	127 (0.02)	0.005	151 (0.03)	126 (0.03)	0.003
Diseases of the arteries, arterioles, and capillaries (%)						
I70	128,683 (6.07)	87,824 (3.20)	0.137	90,566 (4.81)	85,654 (4.55)	0.012
I72	3030 (0.14)	2515 (0.09)	0.015	2367 (0.13)	2296 (0.12)	0.001
I73	65,177 (3.07)	44,425 (1.62)	0.096	47,241 (2.51)	41,939 (2.23)	0.019
I74	4186 (0.20)	2944 (0.11)	0.023	3070 (0.16)	2742 (0.15)	0.004
I77	8600 (0.41)	6471 (0.24)	0.030	6278 (0.33)	6054 (0.32)	0.002
Pulmonary edema (%)						
J81	1062 (0.05)	658 (0.02)	0.014	698 (0.04)	643 (0.03)	0.002
Vascular diseases of the intestine (%)						
K55	673 (0.03)	497 (0.02)	0.009	496 (0.03)	421 (0.02)	0.003

Abbreviations: CCI, Charlson comorbidity index; CVD, cardiovascular diseases; FQ, fluoroquinolones; MAC, macrolides; SD, standard deviation.

After propensity score matching, 1,881,918 episodes of each FQ and macrolide were included in the analyses. Study population characteristics were well balanced (all standardized mean differences <0.2) as displayed in Table 1. The matching approach was applied to all active comparators selected. After matching, the best balancing was achieved in the FQ versus penicillin with beta‐lactamase inhibitors, sulfonamide and trimethoprim combinations, and tetracycline cohorts. The FQ versus lincosamides study cohort had the least preferable balance of baseline characteristics, but balance was still sufficient (see Tables S1a–f).

In the FQ versus macrolides cohort, we identified 934 incident diagnoses of an aortic aneurysm or dissection, of which 599 cases occurred during FQ episodes. This results in a crude incidence rate of 4.20 aortic aneurysm or dissection cases per 10,000 person‐years during FQ episodes compared to 2.24 aortic aneurysm or dissection cases per 10,000 person‐years during macrolide episodes. The age‐ and gender‐standardized incidence rate was 3.10 aortic aneurysm or dissection cases per 10,000 person‐years during FQ episodes compared to 1.88 aortic aneurysm or dissection cases per 10,000 person‐years during macrolide episodes. By applying a multivariate Cox proportional hazard regression model, we estimated a 52% increased risk for aortic aneurysm or dissection within 60 days after FQ prescription compared to a macrolide prescription (Table 2). In the sensitivity analysis with extended follow‐up time to 90 days, the FQ‐associated risk for aortic aneurysm or dissection remained elevated.

**TABLE 2:** Results from the Cox regression, FQ vs. macrolides, stratified by follow‐up duration.

	Follow‐up duration			
	60 days		90 days	
	aHR	[95% CI]	aHR	[95% CI]
FQ episode (ref. macrolides)	1.520	[1.329; 1.738]	1.427	[1.268; 1.605]
Age in years	1.065	[1.060; 1.071]	1.065	[1.060; 1.070]
Males (ref. females)	3.823	[3.315; 4.410]	3.806	[3.356; 4.317]
CCI (ref. 0)				
1–2	1.100	[0.948; 1.275]	1.125	[0.987; 1.283]
3–4	1.171	[0.958; 1.430]	1.186	[0.994; 1.416]
5+	0.872	[0.612; 1.241]	0.936	[0.689; 1.270]
CVD	1.514	[1.293; 1.773]	1.529	[1.330; 1.758]
Diabetes mellitus	0.833	[0.719; 0.965]	0.839	[0.737; 0.955]

Abbreviations: AC, active comparator; aHR, Adjusted hazard ratio; CCI, Charlson comorbidity index; CI, 95% confidence interval [lower bound; bound]; CVD, cardiovascular disease; FQ, fluoroquinolone; ref., reference.

**FIGURE 3:**
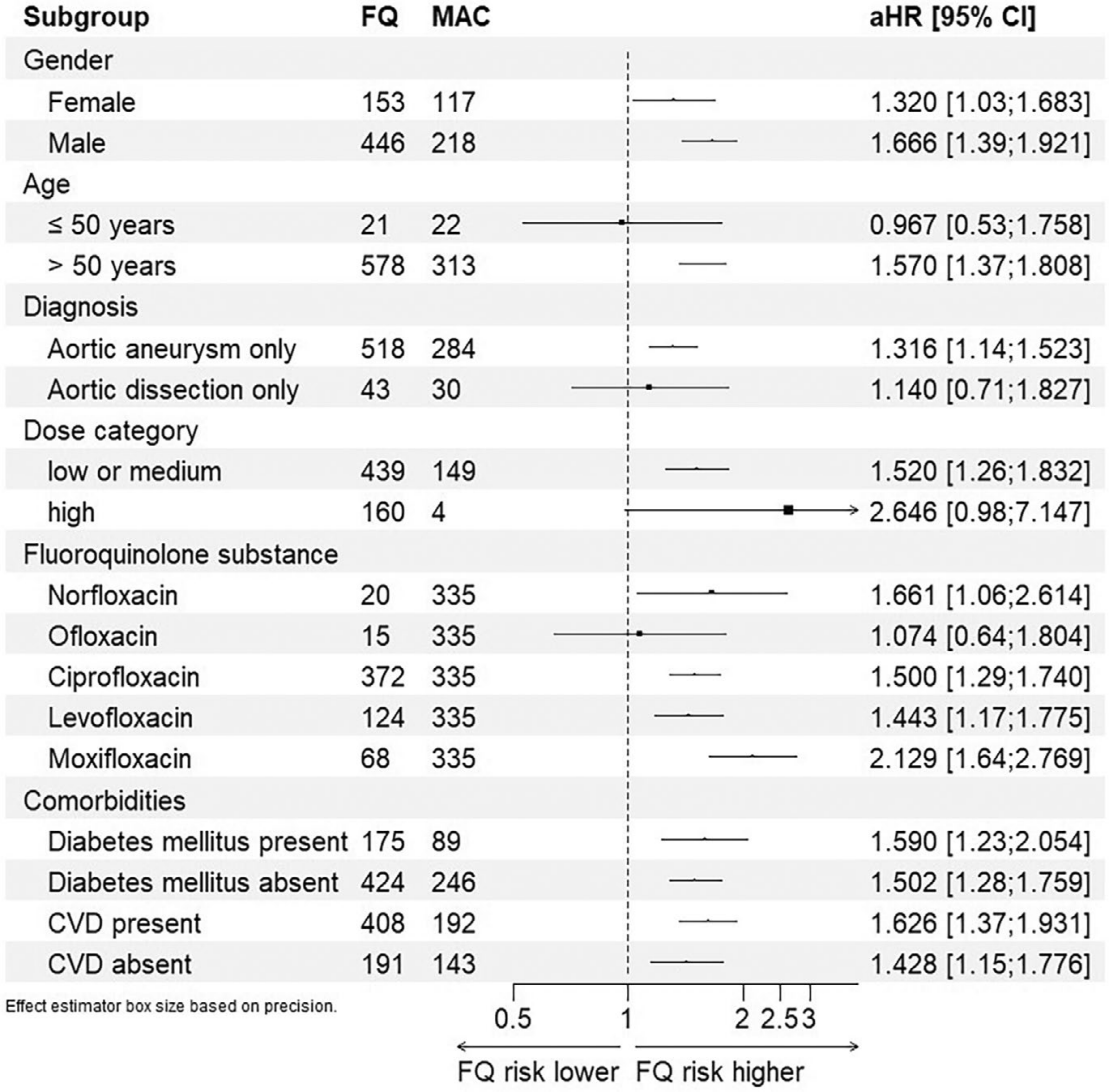
Results from the Cox regression model subgroup analyses.

Figure 3 illustrates the results from subgroup analyses on the risk of aortic aneurysm or dissection associated with FQ episodes compared to macrolide episodes. Relative risks of FQ‐associated aortic aneurysm or dissection were increased for both genders but higher for males. Most cases of aortic aneurysm or dissection occurred at an older age. In this age group (>50 years), FQ episodes were associated with a 57% increased risk of aortic aneurysm or dissection compared to macrolide episodes in the same age group. Due to the small number of aortic aneurysm or dissection events in ≤50‐year‐old individuals, risk estimation was imprecise. Considering only diagnoses of aortic aneurysms, the relative risk was 32% higher during FQ episodes. Furthermore, FQ‐associated risk was higher in the high DDD subgroup compared to the low or medium DDD subgroup. The disaggregation of single FQ substance episodes resulted in an increased risk of aortic aneurysm or dissection during norfloxacin, ciprofloxacin, levofloxacin, and moxifloxacin episodes. Moxifloxacin episodes comprised a 2‐fold increased risk compared to macrolide episodes. For enoxacin, the sample size was too small for multivariate analysis. For ofloxacin, as well as for aortic dissections only, nominally increased risks were found, although 95% CI overlapped 1 (indicating no increased risk); however, these signals with less precise 95% CIs could also be power‐related due to the small number of events included. Moreover, high risks of aortic aneurysm or dissection for FQ episodes were found regardless of the presence of comorbidities (diabetes, cardiovascular diseases).

Table 3 shows the results from multivariate Cox regression models using different active comparators. The relative risks of aortic aneurysm or dissection were also increased for FQ episodes when compared to tetracyclines, penicillins with extended spectrum, second‐ and third‐generation cephalosporins, and lincosamides. Compared to penicillin and beta‐lactamase inhibitor combinations as well as sulfonamide and trimethoprim combinations, the relative risks for FQ‐associated aortic aneurysm or dissection were nominally decreased, but the 95% CI overlapped 1.

**TABLE 3:** Results from the Cox regression, main models with other active comparators.

	Aortic aneurysm/dissection	
	aHR	[95% CI]
FQ vs. tetracyclines	1.859	[1.542; 2.240]
FQ vs. penicillins with extended spectrum	1.451	[1.275; 1.650]
FQ vs. penicillins and beta‐lactamase inhibitors	0.898	[0.764; 1.056]
FQ vs. second‐ and third‐generation cephalosporins	1.229	[1.104; 1.367]
FQ vs. sulfonamide and trimethoprim	0.909	[0.717; 1.153]
FQ vs. lincosamides	1.737	[1.430; 2.110]

*Note*: Regressions were adjusted for age, gender, Charlson comorbidity index, cardiovascular diseases, and diabetes mellitus.

Abbreviations: aHR, adjusted hazard ratio; CI, 95% confidence interval [lower bound; upper bound]; FQ, fluoroquinolone.

Sensitivity analyses regarding unobserved heterogeneity, introduced, for example, by missing information on indication, displayed that there is no evidence for post‐baseline imbalances (such as expected by unobserved heterogeneity) as the variance of random effects was small for all comparisons (Tables S2 and S3).

## 4 DISCUSSION

This population‐based propensity score‐matched cohort study of German adults provides real‐world evidence for FQ‐associated aortic aneurysm or dissection during a 60‐day follow‐up period. The association was primarily driven by aortic aneurysm as the outcome and for ciprofloxacin, levofloxacin, and moxifloxacin as exposure FQ drugs compared to macrolides as active comparators. A dose–response relationship was found, and results remained consistent among all subgroup and sensitivity analyses. Furthermore, the risk of FQ‐associated aortic aneurysm or dissection was also present compared to other antibiotic classes as active comparators. Overall, our results confirm the general conclusion of Garg et al.^6^ that FQ should be used with caution in adults with certain pre‐existing risk factors but additionally demonstrate that antibiotics used for similar indications as FQ are not a homogeneous group and that the choice of the comparison antibiotic may have a pronounced effect on the relative risks associated with FQ.

We were able to replicate the study design and the results of almost all main, subgroup, and sensitivity analyses reported in the MarketScan and Medicare data‐based study,^6^ but with numerically higher relative risks estimated in our German population. In the United States data analysis, aHR was 1.34 [1.17; 1.54] compared to 1.52 [1.33; 1.74] in the German data analysis. This increase might be explainable due to the small modifications applied to the data analysis based on the differences in the available data set itself. Moreover, differences in United States and German health insurance populations and public health services are expected. In Germany there is, for example, a routine screening program of abdominal aortic aneurysms in ≥65‐year‐old male individuals.^21^ In contrast, in the United States, there is only a recommendation of an examination in ≥65‐year‐old male individuals with certain risk factors for aortic aneurysm or dissection.^22^ The fact that the screening routine in Germany is only applicable for male individuals may have contributed to the observed risk differences between males and females.

Although Garg et al.^6^ reported inconclusive results for moxifloxacin (aHR = 0.94 [0.64; 1.38]), which could be attributed to their smaller sample size, we were able to contribute evidence to a high risk of moxifloxacin‐associated aortic aneurysm or dissection with a sufficient number of events (aHR = 2.13 [1.64; 2.77]). In their study, Garg et al.^6^ chose macrolides as the single active comparators, as those drugs are used for similar infections as FQs, and there has not been any evidence linking macrolides to aortic aneurysms and dissections. In German statutory health insurance data, ambulatory diagnoses are available only on a quarterly basis, and patients with a diagnosis of aortic aneurysm or dissection during the quarter of the index prescription have been excluded in order to eliminate cases where the event may have been preexisting before the antibiotic prescription. However, if these inconclusive cases were truly new events, this would exclude a large number of cases and may explain why absolute incidence rates of aortic aneurysm or dissection in this study (4.20 and 2.24 cases per 10,000 person‐years during FQ or macrolide episodes, respectively) were lower than in the Garg et al.^6^ study (19 and 12 cases per 10,000 person‐years, respectively). Nevertheless, since this exclusion criterion was applied to all antibiotic groups (FQ as well as macrolides), one may assume that the relative risks (HR) between FQ exposure and macrolide exposure would be uncompromised by excluding these inconclusive cases with ambulatory diagnoses during the index quarter. German statutory health insurances do not comprise the indication of an antibiotic prescription, and ambulatory diagnoses are only available on a quarterly basis, whereby a direct linkage between infectious diseases with a corresponding antibiotic prescription was not possible. Confounding by indication should therefore be taken into account since an infectious disease itself may be a risk factor for the development of aortic aneurysm or dissection. Moreover, those infectious diseases, such as pneumonia, could increase the possibility of incidentally diagnosing aortic aneurysm or dissection due to X‐ray imaging. Therefore, we applied a frailty model to address unobserved heterogeneity and the resulting post‐baseline imbalances between matched pairs. The variance of random effects was small; we therefore saw no difference between the matched pairs, so there is no evidence for post‐baseline imbalances between matched pairs in our analysis.

After replicating the results of Garg et al.^6^ for macrolides, we extended their work and also compared FQs to other active comparator antibiotics that are also prescribed for similar infections as FQs and for which no evidence for aortic aneurysms and dissections exists. We were able to show that the association between FQ and the outcome differs depending on the chosen AC. For tetracyclines, cephalosporins, penicillins with extended spectrum, and lincosamides, aHRs were similar to the comparison of FQs with macrolides. However, for penicillins in combination with beta‐lactamase inhibitors as well as for sulfonamide and trimethoprim combinations, results were inconclusive, and the estimated aHR and lower bound of 95% CIs indicated a protective effect for FQ episodes compared to active comparator episodes. An increased relative risk of FQ was also reported by other previous studies based on US claims data such as comparing FQ to azithromycin and amoxicillin^9^ or to a variety of active comparators.^8^ Likewise, a Swedish cohort study^10^ reported an increased relative risk for FQ‐associated aortic aneurysm or dissection compared to amoxicillin.

To the best of our knowledge, this is the first large‐scale cohort study based on German data comparing FQ to several active comparators separately addressing the outcome of aortic aneurysm or dissection. We further confirmed the increased risk of aortic aneurysm or dissection associated with FQ use, which should be taken into account by physicians when prescribing antibiotics. By using an active comparator new user design, we have reduced the risk of confounding by indication since comparable antibiotics were used as reference groups. By analyzing several active comparators separately, we provided detailed information on differences between antibiotics. We additionally minimized confounding by baseline conditions and the problem of non‐random treatment allocation by applying a propensity score matching approach. Our data cover one‐third of the German population^15^ and offer population‐based insights into the real‐world health care situation of a representative patient population in Germany. Thus, we were able to conduct a large‐scale cohort study with a high number of aortic aneurysm or dissection events. As claims data are used primarily for billing purposes, we expect a consistently high degree of completeness in all diagnoses and drug prescriptions used to create our outcome, exposure, and covariates. As health care access within the statutory health system in Germany is not limited by financial constraints, we also expect with a high degree of certainty that all relevant cases have been recorded.

In conclusion, in this German cohort study, a 52% increased risk of aortic aneurysm or dissection associated with FQ episodes compared to macrolide episodes was observed. The relative risk of FQ episodes increased up to 86% depending on the active comparator chosen for the analysis. Risk estimates remained consistent across subgroup and sensitivity analyses. High‐risk groups observed were male individuals, individuals aged >50 years, and individuals with a high daily dose. These findings confirm that FQ should be prescribed with caution, and alternative first‐line antibiotics should be preferred if possible.

## AUTHOR CONTRIBUTIONS

All authors conceived and designed the study. KS, GB, AS, and HS had full access to all the data in the study and take full responsibility for the integrity of the data set provided for the statistical analysis. JW, JP, CB, and BH had full access to the statistical analysis data set and carried out the statistical analysis. JW and JP analyzed the data. All authors interpreted the data and critically revised the manuscript for important intellectual content. JW drafted the manuscript. JW and BH are the guarantors.

## CONFLICT OF INTEREST STATEMENT

The authors declare no conflicts of interest. All authors declare no support from any organization for the submitted work; and no financial relationships with any organizations that might have an interest in the submitted work in the previous 3 years; no other relationships or activities that could appear to have influenced the submitted work.

## Supporting information


Appendix S1.

## Data Availability

No additional data available. Statistical code available on request. The manuscript's guarantors affirm that the manuscript is an honest, accurate, and transparent account of the study being reported; that no important aspects of the study have been omitted; and that any discrepancies from the study as planned have been explained.

## References

[1] Ezelarab HAA , Abbas SH , Hassan HA , Abuo‐Rahma GEA . Recent updates of fluoroquinolones as antibacterial agents. Arch Pharm. 2018;351(9):e1800141. doi:10.1002/ardp.201800141 30048015

[2] European Medicines Agency . Disabling and potentially permanent side effects lead to suspension or restrictions of quinolone and fluoroquinolone antibiotics. EMA/175398/2019. 2019. Accessed May 28, 2024. https://www.ema.europa.eu/en/documents/referral/quinolone‐and‐fluoroquinolone‐article‐31‐referral‐disabling‐and‐potentially‐permanent‐side‐effects‐lead‐suspension‐or‐restrictions‐quinolone‐and‐fluoroquinolone‐antibiotics_en.pdf

[3] Ly NF , Flach C , Lysen TS , et al. Impact of European Union label changes for fluoroquinolone‐containing medicinal products for systemic and inhalation use: post‐referral prescribing trends. Drug Saf. 2023;46(4):405‐416. doi:10.1007/s40264-023-01286-4 36976448 PMC10044099

[4] Guzik B , Sagan A , Ludew D , et al. Mechanisms of oxidative stress in human aortic aneurysms–association with clinical risk factors for atherosclerosis and disease severity. Int J Cardiol. 2013;168(3):2389‐2396. doi:10.1016/j.ijcard.2013.01.278 23506637 PMC3819986

[5] Guzzardi DG , Teng G , Kang S , et al. Induction of human aortic myofibroblast‐mediated extracellular matrix dysregulation: a potential mechanism of fluoroquinolone‐associated aortopathy. J Thorac Cardiovasc Surg. 2019;157(1):109‐119.e2. doi:10.1016/j.jtcvs.2018.08.079 30528439

[6] Garg M , Venugopalan V , Vouri SM , Diaby V , Iovine NM , Park H . Oral fluoroquinolones and risk of aortic aneurysm or dissection: a nationwide population‐based propensity score‐matched cohort study. Pharmacotherapy. 2023;43(9):883‐893. doi:10.1002/phar.2841 37381584

[7] Chen YY , Yang SF , Yeh HW , et al. Association between aortic aneurysm and aortic dissection with fluoroquinolones use in patients with urinary tract infections: a population‐based cohort study. J Am Heart Assoc. 2022;11(6):e023267. doi:10.1161/jaha.121.023267 35229623 PMC9075302

[8] Newton ER , Akerman AW , Strassle PD , Kibbe MR . Association of fluoroquinolone use with short‐term risk of development of aortic aneurysm. JAMA Surg. 2021;156(3):264‐272. doi:10.1001/jamasurg.2020.6165 33404647 PMC7788511

[9] Gopalakrishnan C , Bykov K , Fischer MA , Connolly JG , Gagne JJ , Fralick M . Association of Fluoroquinolones with the risk of aortic aneurysm or aortic dissection. JAMA Intern Med. 2020;180(12):1596‐1605. doi:10.1001/jamainternmed.2020.4199 32897307 PMC7489402

[10] Pasternak B , Inghammar M , Svanström H . Fluoroquinolone use and risk of aortic aneurysm and dissection: nationwide cohort study. BMJ. 2018;360:k678. doi:10.1136/bmj.k678 29519881 PMC5842359

[11] Lee CC , Lee MT , Chen YS , Lee SH , Chen SC , Chang SC . Risk of aortic dissection and aortic aneurysm in patients taking oral fluoroquinolone. JAMA Intern Med. 2015;175(11):1839‐1847. doi:10.1001/jamainternmed.2015.5389 26436523

[12] Daneman N , Lu H , Redelmeier DA . Fluoroquinolones and collagen associated severe adverse events: a longitudinal cohort study. BMJ Open. 2015;5(11):e010077. doi:10.1136/bmjopen-2015-010077 PMC4654346 26582407

[13] Son N , Choi E , Chung SY , Han SY , Kim B . Risk of aortic aneurysm and aortic dissection with the use of fluoroquinolones in Korea: a nested case‐control study. BMC Cardiovasc Disord. 2022;22(1):44. doi:10.1186/s12872-022-02488-x 35152888 PMC8842902

[14] Lawaetz Kristensen K , Hallas J , Sanddal Lindholt J . Fluoroquinolones as a trigger for rupture of abdominal aortic aneurysm: a case‐crossover analysis. Basic Clin Pharmacol Toxicol. 2021;129(1):44‐51. doi:10.1111/bcpt.13591 33887112

[15] Bundesministerium für Gesundheit (BMG) . Gesetzliche Krankenversicherung ‐ Mitglieder, mitversicherte Angehörige und Krankenstand 2024. Accessed January 30, 2024. https://www.bundesgesundheitsministerium.de/themen/krankenversicherung/zahlen‐und‐fakten‐zur‐krankenversicherung/mitglieder‐und‐versicherte.html

[16] Bundesinstitut für Arzneimittel und Medizinprodukte (BfArM) . Classifications. Accessed May 28, 2024. https://www.bfarm.de/EN/Code‐systems/Classifications/_node.html

[17] Schneeweiss S , Rassen JA , Brown JS , et al. Graphical depiction of longitudinal study designs in health care databases. Ann Intern Med. 2019;170(6):398‐406. doi:10.7326/m18-3079 30856654

[18] Charlson ME , Pompei P , Ales KL , MacKenzie CR . A new method of classifying prognostic comorbidity in longitudinal studies: development and validation. J Chronic Dis. 1987;40(5):373‐383. doi:10.1016/0021-9681(87)90171-8 3558716

[19] Quan H , Li B , Couris CM , et al. Updating and validating the Charlson comorbidity index and score for risk adjustment in hospital discharge abstracts using data from 6 countries. Am J Epidemiol. 2011;173(6):676‐682. doi:10.1093/aje/kwq433 21330339

[20] Statistisches Bundesamt (DeStatis) . Bevölkerung zum Stichtag 31.12. des jeweiligen Jahres. Gliederungsmerkmale: Jahre, Region, Alter, Geschlecht, Nationalität (Grundlage Zensus 2011). Accessed January 30, 2025. https://www.gbe‐bund.de/gbe/isgbe.suche?p_uid=gast&p_aid=12375960&p_knoten=VR&p_sprache=D&p_adv_search=&p_methode=2&p_volltext=1&p_synonyme=1&p_soundex=&p_suchstring=Bev%C3%B6lkerung%20zum%20Stichtag%2031.12

[21] Kassenärztliche Bundesvereinigung . Früherkennung auf Bauchaortenaneurysmen. Accessed August 01, 2024. https://www.kbv.de/html/34733.php#:~:text=Neue%20Vorsorgeuntersuchung%20f%C3%BCr%20M%C3%A4nner%20ab,Fr%C3%BCherkennung%20von%20Aneurysmen%20der%20Bauchschlagader

[22] U.S. Preventice Service Task Force . Abdominal Aortic Aneurysm: Screening. Accessed August 01, 2024. https://www.uspreventiveservicestaskforce.org/uspstf/recommendation/abdominal‐aortic‐aneurysm‐screening

