# Supplementary material for: Fluoroquinolones and the risk of aortic aneurysm or dissection: A population‐based propensity score‐matched German cohort study
Source: Pharmacotherapy. 2025 Apr 26;45(6):314–23. doi: 10.1002/phar.70020 (PMC12149785; doi:10.1002/phar.70020)
Supplement: Supplementary file 1 — Appendix S1. [file PHAR-45-314-s001.docx]

eTable 1a. Study population characteristics before and after propensity score matching, cohort FQ vs tetracyclines.

|  | | **Before Propensity Score Matching** | |  |  | **After Propensity Score Matching** | |  |
| --- | --- | --- | --- | --- | --- | --- | --- | --- |
|  | | **FQ**  ***(n = 2,121,502)*** | **TETRA  *(n = 1,337,495)*** | **Standardized difference** |  | **FQ  *(n = 1,312,680)*** | **TETRA *(n = 1,312,680)*** | **Standardized difference** |
| Age (mean (SD)) | | 56.51 (19.32) | 49.47 (18.53) | 0.372 |  | 49.99 (18.45) | 49.90 (18.43) | 0.004 |
| Male gender (%) | | 868,490 (40.94) | 648,784 (48.51) | 0.153 |  | 604,660 (46.06) | 623,973 (47.53) | 0.029 |
| CCI (%) | |  |  | 0.190 |  |  |  | 0.007 |
| 0 | | 1,295,906 (61.08) | 927,134 (69.32) |  |  | 906,560 (69.06) | 902,340 (68.74) |  |
| 1-2 | | 615,584 (29.02) | 328,305 (24.55) |  |  | 325,316 (24.78) | 328,288 (25.01) |  |
| 3-4 | | 162,410 (7.66) | 65,581 (4.90) |  |  | 64,879 (4.94) | 65,577 (5.00) |  |
| 5+ | | 47,602 (2.24) | 16,475 (1.23) |  |  | 15,925 (1.21) | 16,475 (1.26) |  |
| Drugs dispensed (%) | |  |  | 0.292 |  |  |  | 0.020 |
| 0 | | 330,966 (15.60) | 284,239 (21.25) |  |  | 268,512 (20.46) | 267,430 (20.37) |  |
| 1-3 | | 436,333 (20.57) | 350,325 (26.19) |  |  | 341,152 (25.99) | 350,311 (26.69) |  |
| 4-10 | | 518,373 (24.43) | 339,330 (25.37) |  |  | 340,585 (25.95) | 331,340 (25.24) |  |
| 11-20 | | 382,195 (18.02) | 194,190 (14.52) |  |  | 194,329 (14.80) | 194,188 (14.79) |  |
| 21+ | | 453,635 (21.38) | 169,411 (12.67) |  |  | 168,102 (12.81) | 169,411 (12.91) |  |
| Diabetes mellitus (%) | E10-14 | 410,669 (19.36) | 174,523 (13.05) | 0.172 |  | 174,039 (13.26) | 174,511 (13.29) | 0.001 |
| Cerebral vascular syndromes (%) | G45 | 19,816 (0.93) | 7,810 (0.58) | 0.040 |  | 7,083 (0.54) | 7,810 (0.60) | 0.007 |
|  | G46 | 1,345 (0.06) | 544 (0.04) | 0.010 |  | 474 (0.04) | 544 (0.04) | 0.003 |
| Hypertension (%) | I11 | 117,528 (5.54) | 48,916 (3.66) | 0.090 |  | 46,648 (3.55) | 48,912 (3.73) | 0.009 |
|  | I13 | 4,425 (0.21) | 1,489 (0.11) | 0.024 |  | 1,303 (0.10) | 1,489 (0.11) | 0.004 |
| Ischemic heart disease (%) | I20 | 38,748 (1.83) | 18,296 (1.37) | 0.037 |  | 16,851 (1.28) | 18,269 (1.39) | 0.009 |
|  | I22 | 472 (0.02) | 225 (0.02) | 0.004 |  | 187 (0.01) | 225 (0.02) | 0.002 |
|  | I24 | 4,879 (0.23) | 1,962 (0.15) | 0.019 |  | 1,685 (0.13) | 1,962 (0.15) | 0.006 |
|  | I25 | 247,471 (11.67) | 98,340 (7.35) | 0.147 |  | 95,134 (7.25) | 98,339 (7.49) | 0.009 |
| Valve diseases (%) | I34 | 70,978 (3.35) | 30,203 (2.26) | 0.066 |  | 27,252 (2.08) | 30,189 (2.30) | 0.015 |
|  | I35 | 57,298 (2.70) | 22,960 (1.72) | 0.067 |  | 20,806 (1.59) | 22,957 (1.75) | 0.013 |
|  | I36 | 7,498 (0.35) | 3,239 (0.24) | 0.020 |  | 2,679 (0.20) | 3,239 (0.25) | 0.009 |
|  | I37 | 2,440 (0.12) | 1,213 (0.09) | 0.008 |  | 1,051 (0.08) | 1,210 (0.09) | 0.004 |
| Cardiomyopathy (%) | I42 | 18,740 (0.88) | 8,184 (0.61) | 0.032 |  | 7,109 (0.54) | 8,181 (0.62) | 0.011 |
|  | I43 | 959 (0.05) | 498 (0.04) | 0.004 |  | 421 (0.03) | 498 (0.04) | 0.003 |
| Cardiac arrhythmia (%) | I44 | 33,728 (1.59) | 13,565 (1.01) | 0.051 |  | 12,332 (0.94) | 13,565 (1.03) | 0.010 |
|  | I45 | 21,861 (1.03) | 10,540 (0.79) | 0.026 |  | 9,530 (0.73) | 10,502 (0.80) | 0.009 |
|  | I46 | 481 (0.02) | 217 (0.02) | 0.005 |  | 169 (0.01) | 217 (0.02) | 0.003 |
|  | I47 | 24,512 (1.16) | 12,444 (0.93) | 0.022 |  | 11,400 (0.87) | 12,441 (0.95) | 0.008 |
|  | I48 | 105,150 (4.96) | 36,751 (2.75) | 0.115 |  | 34,580 (2.63) | 36,750 (2.80) | 0.010 |
|  | I49 | 129,689 (6.11) | 59,408 (4.44) | 0.075 |  | 56,543 (4.31) | 59,388 (4.52) | 0.011 |
| Heart failure (%) | I50 | 152,715 (7.20) | 54,442 (4.07) | 0.136 |  | 51,969 (3.96) | 54,441 (4.15) | 0.010 |
| Cerebrovascular diseases (%) | I61 | 3,139 (0.15) | 1,127 (0.08) | 0.019 |  | 938 (0.07) | 1,127 (0.09) | 0.005 |
|  | I62 | 1,034 (0.05) | 434 (0.03) | 0.008 |  | 353 (0.03) | 434 (0.03) | 0.004 |
|  | I63 | 21,214 (1.00) | 7,532 (0.56) | 0.050 |  | 6,733 (0.51) | 7,531 (0.57) | 0.008 |
|  | I64 | 20,752 (0.98) | 6,828 (0.51) | 0.054 |  | 6,230 (0.48) | 6,828 (0.52) | 0.006 |
|  | I65 | 54,031 (2.55) | 21,953 (1.64) | 0.063 |  | 20,210 (1.54) | 21,951 (1.67) | 0.011 |
|  | I66 | 2,550 (0.12) | 1,061 (0.08) | 0.013 |  | 886 (0.07) | 1,061 (0.08) | 0.005 |
|  | I67 | 71,670 (3.38) | 27,473 (2.05) | 0.082 |  | 25,704 (1.96) | 27,473 (2.09) | 0.010 |
|  | I68 | 215 (0.03) | 73 (0.02) | 0.004 |  | 64 (0.02) | 73 (0.02) | 0.002 |
| Diseases of the arteries, arterioles and capillaries (%) | I70 | 128,683 (6.07) | 51,268 (3.83) | 0.103 |  | 48,954 (3.73) | 51,266 (3.91) | 0.009 |
|  | I72 | 3,030 (0.14) | 1,320 (0.10) | 0.013 |  | 1,175 (0.09) | 1,320 (0.10) | 0.004 |
|  | I73 | 65,177 (3.07) | 26,102 (1.95) | 0.072 |  | 25,000 (1.91) | 26,098 (1.99) | 0.006 |
|  | I74 | 4,186 (0.20) | 1,694 (0.13) | 0.018 |  | 1,500 (0.11) | 1,694 (0.13) | 0.004 |
|  | I77 | 8,600 (0.41) | 3,595 (0.27) | 0.024 |  | 3,195 (0.24) | 3,595 (0.27) | 0.006 |
| Pulmonary edema (%) | J81 | 1,062 (0.05) | 347 (0.03) | 0.012 |  | 261 (0.02) | 347 (0.03) | 0.004 |
| Vascular diseases of the intestine (%) | K55 | 673 (0.03) | 258 (0.02) | 0.008 |  | 225 (0.02) | 258 (0.02) | 0.002 |
| Fluoroquinolones (FQ) \| tetracyclines (TETRA) \| standard deviation (SD) \| Charlson comorbidity index (CCI) \| cardiovascular diseases (CVD). | | | | | | | | |

eTable 1b. Study population characteristics before and after propensity score matching, cohort FQ vs penicillins with extended spectrum.

|  | | **Before Propensity Score Matching** | |  |  | **After Propensity Score Matching** | |  |
| --- | --- | --- | --- | --- | --- | --- | --- | --- |
|  | | **FQ**  ***(n = 2,121,502)*** | **PEN *(n = 2,705,063)*** | **Standardized difference** |  | **FQ *(n = 1,888,401)*** | **PEN *(n = 1,888,401)*** | **Standardized difference** |
| Age (mean (SD)) | | 56.51 (19.32) | 47.13 (18.26) | 0.499 |  | 53.83 (18.65) | 52.86 (17.90) | 0.053 |
| Male gender (%) | | 868,490 (40.94) | 1,318,576 (48.75) | 0.157 |  | 829,728 (43.94) | 791,372 (41.91) | 0.041 |
| CCI (%) | |  |  | 0.228 |  |  |  | 0.059 |
| 0 | | 1,295,906 (61.08) | 1,920,427 (70.99) |  |  | 1,198,433 (63.46) | 1,231,865 (65.23) |  |
| 1-2 | | 615,584 (29.02) | 633,647 (23.42) |  |  | 518,136 (27.44) | 513,088 (27.17) |  |
| 3-4 | | 162,410 (7.66) | 121,036 (4.47) |  |  | 133,013 (7.04) | 114,098 (6.04) |  |
| 5+ | | 47,602 (2.24) | 29,953 (1.11) |  |  | 38,819 (2.06) | 29,350 (1.55) |  |
| Drugs dispensed (%) | |  |  | 0.366 |  |  |  | 0.059 |
| 0 | | 330,966 (15.60) | 668,321 (24.71) |  |  | 329,594 (17.45) | 334,411 (17.71) |  |
| 1-3 | | 436,333 (20.57) | 735,589 (27.19) |  |  | 432,932 (22.93) | 437,626 (23.17) |  |
| 4-10 | | 518,373 (24.43) | 630,057 (23.29) |  |  | 459,043 (24.31) | 490,874 (25.99) |  |
| 11-20 | | 382,195 (18.02) | 344,221 (12.73) |  |  | 315,272 (16.70) | 312,112 (16.53) |  |
| 21+ | | 453,635 (21.38) | 326,875 (12.08) |  |  | 351,560 (18.62) | 313,378 (16.60) |  |
| Diabetes mellitus (%) | E10-14 | 410,669 (19.36) | 323,235 (11.95) | 0.205 |  | 329,828 (17.47) | 300,265 (15.90) | 0.042 |
| Cerebral vascular syndromes (%) | G45 | 19,816 (0.93) | 13,942 (0.52) | 0.049 |  | 17,780 (0.94) | 13,301 (0.70) | 0.026 |
|  | G46 | 1,345 (0.06) | 1,048 (0.04) | 0.011 |  | 1,238 (0.07) | 980 (0.05) | 0.006 |
| Hypertension (%) | I11 | 117,528 (5.54) | 84,804 (3.14) | 0.118 |  | 101,526 (5.38) | 81,615 (4.32) | 0.049 |
|  | I13 | 4,425 (0.21) | 2,635 (0.10) | 0.028 |  | 3,863 (0.21) | 2,568 (0.14) | 0.017 |
| Ischemic heart disease (%) | I20 | 38,748 (1.83) | 29,889 (1.11) | 0.060 |  | 34,666 (1.84) | 27,648 (1.46) | 0.029 |
|  | I22 | 472 (0.02) | 407 (0.02) | 0.005 |  | 440 (0.02) | 367 (0.02) | 0.003 |
|  | I24 | 4,879 (0.23) | 3,829 (0.14) | 0.021 |  | 4,542 (0.24) | 3,575 (0.19) | 0.011 |
|  | I25 | 247,471 (11.67) | 176,973 (6.54) | 0.179 |  | 209,299 (11.08) | 170,106 (9.01) | 0.069 |
| Valve diseases (%) | I34 | 70,978 (3.35) | 62,645 (2.32) | 0.062 |  | 66,445 (3.52) | 55,933 (2.96) | 0.031 |
|  | I35 | 57,298 (2.70) | 53,547 (1.98) | 0.048 |  | 53,684 (2.84) | 46,955 (2.49) | 0.022 |
|  | I36 | 7,498 (0.35) | 6,454 (0.24) | 0.021 |  | 7,070 (0.37) | 5,770 (0.31) | 0.012 |
|  | I37 | 2,440 (0.12) | 2,790 (0.10) | 0.004 |  | 2,337 (0.12) | 1,905 (0.10) | 0.007 |
| Cardiomyopathy (%) | I42 | 18,740 (0.88) | 17,269 (0.64) | 0.028 |  | 17,798 (0.94) | 15,057 (0.80) | 0.016 |
|  | I43 | 959 (0.05) | 595 (0.02) | 0.013 |  | 856 (0.05) | 572 (0.03) | 0.008 |
| Cardiac arrhythmia (%) | I44 | 33,728 (1.59) | 26,427 (0.98) | 0.054 |  | 30,617 (1.62) | 24,511 (1.30) | 0.027 |
|  | I45 | 21,861 (1.03) | 20,387 (0.75) | 0.029 |  | 20,639 (1.09) | 17,326 (0.92) | 0.018 |
|  | I46 | 481 (0.02) | 445 (0.02) | 0.004 |  | 453 (0.02) | 364 (0.02) | 0.003 |
|  | I47 | 24,512 (1.16) | 21,179 (0.78) | 0.038 |  | 22,552 (1.19) | 18,956 (1.00) | 0.018 |
|  | I48 | 105,150 (4.96) | 73,701 (2.73) | 0.116 |  | 92,390 (4.89) | 71,022 (3.76) | 0.056 |
|  | I49 | 129,689 (6.11) | 101,862 (3.77) | 0.108 |  | 112,692 (5.97) | 93,924 (4.97) | 0.044 |
| Heart failure (%) | I50 | 152,715 (7.20) | 101,080 (3.74) | 0.153 |  | 126,169 (6.68) | 97,385 (5.16) | 0.065 |
| Cerebrovascular diseases (%) | I61 | 3,139 (0.15) | 2,288 (0.09) | 0.019 |  | 2,784 (0.15) | 2,118 (0.11) | 0.010 |
|  | I62 | 1,034 (0.05) | 806 (0.03) | 0.010 |  | 948 (0.05) | 701 (0.04) | 0.006 |
|  | I63 | 21,214 (1.00) | 14,428 (0.53) | 0.054 |  | 18,821 (1.00) | 13,872 (0.74) | 0.028 |
|  | I64 | 20,752 (0.98) | 13,445 (0.50) | 0.056 |  | 18,403 (0.98) | 13,101 (0.69) | 0.031 |
|  | I65 | 54,031 (2.55) | 40,295 (1.49) | 0.075 |  | 47,294 (2.50) | 39,052 (2.07) | 0.029 |
|  | I66 | 2,550 (0.12) | 2,015 (0.07) | 0.015 |  | 2,372 (0.13) | 1,838 (0.10) | 0.008 |
|  | I67 | 71,670 (3.38) | 46,579 (1.72) | 0.105 |  | 60,247 (3.19) | 45,528 (2.41) | 0.047 |
|  | I68 | 215 (0.03) | 152 (0.03) | 0.002 |  | 166 (0.03) | 142 (0.03) | <0.001 |
| Diseases of the arteries, arterioles and capillaries (%) | I70 | 128,683 (6.07) | 91,292 (3.38) | 0.127 |  | 111,120 (5.88) | 88,776 (4.70) | 0.053 |
|  | I72 | 3,030 (0.14) | 2,561 (0.10) | 0.014 |  | 2,831 (0.15) | 2,321 (0.12) | 0.007 |
|  | I73 | 65,177 (3.07) | 45,771 (1.69) | 0.091 |  | 57,496 (3.05) | 44,189 (2.34) | 0.044 |
|  | I74 | 4,186 (0.20) | 3,250 (0.12) | 0.019 |  | 3,860 (0.20) | 3,011 (0.16) | 0.011 |
|  | I77 | 8,600 (0.41) | 6,750 (0.25) | 0.027 |  | 7,880 (0.42) | 6,298 (0.33) | 0.014 |
| Pulmonary edema (%) | J81 | 1,062 (0.05) | 675 (0.03) | 0.013 |  | 958 (0.05) | 646 (0.03) | 0.008 |
| Vascular diseases of the intestine (%) | K55 | 673 (0.03) | 473 (0.02) | 0.009 |  | 605 (0.03) | 426 (0.02) | 0.006 |
| Fluoroquinolones (FQ) \| penicillins with extended spectrum (PEN) \| standard deviation (SD) \| Charlson comorbidity index (CCI) \| cardiovascular diseases (CVD). | | | | | | | | |

eTable 1c. Study population characteristics before and after propensity score matching, cohort FQ vs penicillins with ß-lactamase inhibitors.

|  | | **Before Propensity Score Matching** | |  |  | **After Propensity Score Matching** | |  |
| --- | --- | --- | --- | --- | --- | --- | --- | --- |
|  | | **FQ**  ***(n = 2,121,502)*** | **PEN BETA *(n = 882,904)*** | **Standardized difference** |  | **FQ *(n = 882,904)*** | **PEN BETA *(n = 882,904)*** | **Standardized difference** |
| Age (mean (SD)) | | 56.51 (19.32) | 52.76 (19.52) | 0.193 |  | 52.67 (19.42) | 52.76 (19.52) | 0.005 |
| Male gender (%) | | 868,490 (40.94) | 455,650 (51.61) | 0.215 |  | 455,643 (51.61) | 455,650 (51.61) | <0.001 |
| CCI (%) | |  |  | 0.051 |  |  |  | 0.007 |
| 0 | | 1,295,906 (61.08) | 560,931 (63.53) |  |  | 563,113 (63.78) | 560,931 (63.53) |  |
| 1-2 | | 615,584 (29.02) | 242,108 (27.42) |  |  | 241,496 (27.35) | 242,108 (27.42) |  |
| 3-4 | | 162,410 (7.66) | 61,597 (6.98) |  |  | 60,475 (6.85) | 61,597 (6.98) |  |
| 5+ | | 47,602 (2.24) | 18,268 (2.07) |  |  | 17,820 (2.02) | 18,268 (2.07) |  |
| Drugs dispensed (%) | |  |  | 0.159 |  |  |  | 0.008 |
| 0 | | 330,966 (15.60) | 182,436 (20.66) |  |  | 183,271 (20.76) | 182,436 (20.66) |  |
| 1-3 | | 436,333 (20.57) | 199,214 (22.56) |  |  | 200,188 (22.67) | 199,214 (22.56) |  |
| 4-10 | | 518,373 (24.43) | 194,848 (22.07) |  |  | 195,824 (22.18) | 194,848 (22.07) |  |
| 11-20 | | 382,195 (18.02) | 133,168 (15.08) |  |  | 133,119 (15.08) | 133,168 (15.08) |  |
| 21+ | | 453,635 (21.38) | 173,238 (19.62) |  |  | 170,502 (19.31) | 173,238 (19.62) |  |
| Diabetes mellitus (%) | E10-14 | 410,669 (19.36) | 150,272 (17.02) | 0.061 |  | 148,466 (16.82) | 150,272 (17.02) | 0.005 |
| Cerebral vascular syndromes (%) | G45 | 19,816 (0.93) | 6,712 (0.76) | 0.019 |  | 6,112 (0.69) | 6,712 (0.76) | 0.008 |
|  | G46 | 1,345 (0.06) | 472 (0.05) | 0.004 |  | 380 (0.04) | 472 (0.05) | 0.005 |
| Hypertension (%) | I11 | 117,528 (5.54) | 42,584 (4.82) | 0.032 |  | 40,257 (4.56) | 42,584 (4.82) | 0.012 |
|  | I13 | 4,425 (0.21) | 1,748 (0.20) | 0.002 |  | 1,447 (0.16) | 1,748 (0.20) | 0.008 |
| Ischemic heart disease (%) | I20 | 38,748 (1.83) | 12,583 (1.43) | 0.032 |  | 11,860 (1.34) | 12,583 (1.43) | 0.007 |
|  | I22 | 472 (0.02) | 182 (0.02) | 0.001 |  | 153 (0.02) | 182 (0.02) | 0.002 |
|  | I24 | 4,879 (0.23) | 1,652 (0.19) | 0.009 |  | 1,415 (0.16) | 1,652 (0.19) | 0.006 |
|  | I25 | 247,471 (11.67) | 87,772 (9.94) | 0.056 |  | 84,209 (9.54) | 87,772 (9.94) | 0.014 |
| Valve diseases (%) | I34 | 70,978 (3.35) | 26,244 (2.97) | 0.021 |  | 23,638 (2.68) | 26,244 (2.97) | 0.018 |
|  | I35 | 57,298 (2.70) | 22,113 (2.51) | 0.012 |  | 19,753 (2.24) | 22,113 (2.51) | 0.018 |
|  | I36 | 7,498 (0.35) | 2,953 (0.33) | 0.003 |  | 2,477 (0.28) | 2,953 (0.33) | 0.010 |
|  | I37 | 2,440 (0.12) | 966 (0.11) | 0.002 |  | 793 (0.09) | 966 (0.11) | 0.006 |
| Cardiomyopathy (%) | I42 | 18,740 (0.88) | 8,256 (0.94) | 0.005 |  | 7,155 (0.81) | 8,256 (0.94) | 0.013 |
|  | I43 | 959 (0.05) | 383 (0.04) | 0.001 |  | 323 (0.04) | 383 (0.04) | 0.003 |
| Cardiac arrhythmia (%) | I44 | 33,728 (1.59) | 12,898 (1.46) | 0.011 |  | 11,536 (1.31) | 12,898 (1.46) | 0.013 |
|  | I45 | 21,861 (1.03) | 8,660 (0.98) | 0.005 |  | 7,879 (0.89) | 8,660 (0.98) | 0.009 |
|  | I46 | 481 (0.02) | 196 (0.02) | <0.001 |  | 159 (0.02) | 196 (0.02) | 0.003 |
|  | I47 | 24,512 (1.16) | 8,545 (0.97) | 0.018 |  | 8,040 (0.91) | 8,545 (0.97) | 0.006 |
|  | I48 | 105,150 (4.96) | 43,711 (4.95) | <0.001 |  | 41,113 (4.66) | 43,711 (4.95) | 0.014 |
|  | I49 | 129,689 (6.11) | 44,300 (5.02) | 0.048 |  | 42,546 (4.82) | 44,300 (5.02) | 0.009 |
| Heart failure (%) | I50 | 152,715 (7.20) | 58,585 (6.64) | 0.022 |  | 56,119 (6.36) | 58,585 (6.64) | 0.011 |
| Cerebrovascular diseases (%) | I61 | 3,139 (0.15) | 1,170 (0.13) | 0.004 |  | 980 (0.11) | 1,170 (0.13) | 0.006 |
|  | I62 | 1,034 (0.05) | 450 (0.05) | 0.001 |  | 377 (0.04) | 450 (0.05) | 0.004 |
|  | I63 | 21,214 (1.00) | 8,116 (0.92) | 0.008 |  | 7,172 (0.81) | 8,116 (0.92) | 0.012 |
|  | I64 | 20,752 (0.98) | 7,308 (0.83) | 0.016 |  | 6,652 (0.75) | 7,308 (0.83) | 0.008 |
|  | I65 | 54,031 (2.55) | 20,545 (2.33) | 0.014 |  | 18,542 (2.10) | 20,545 (2.33) | 0.015 |
|  | I66 | 2,550 (0.12) | 905 (0.10) | 0.005 |  | 746 (0.08) | 905 (0.10) | 0.006 |
|  | I67 | 71,670 (3.38) | 23,958 (2.71) | 0.039 |  | 22,480 (2.55) | 23,958 (2.71) | 0.010 |
|  | I68 | 215 (0.03) | 80 (0.03) | 0.001 |  | 70 (0.03) | 80 (0.03) | 0.002 |
| Diseases of the arteries, arterioles and capillaries (%) | I70 | 128,683 (6.07) | 48,261 (5.47) | 0.026 |  | 46,221 (5.24) | 48,261 (5.47) | 0.010 |
|  | I72 | 3,030 (0.14) | 1,168 (0.13) | 0.003 |  | 1,000 (0.11) | 1,168 (0.13) | 0.005 |
|  | I73 | 65,177 (3.07) | 24,947 (2.83) | 0.015 |  | 23,686 (2.68) | 24,947 (2.83) | 0.009 |
|  | I74 | 4,186 (0.20) | 1,694 (0.19) | 0.001 |  | 1,417 (0.16) | 1,694 (0.19) | 0.007 |
|  | I77 | 8,600 (0.41) | 3,261 (0.37) | 0.006 |  | 2,891 (0.33) | 3,261 (0.37) | 0.007 |
| Pulmonary edema (%) | J81 | 1,062 (0.05) | 452 (0.05) | 0.001 |  | 365 (0.04) | 452 (0.05) | 0.005 |
| Vascular diseases of the intestine (%) | K55 | 673 (0.03) | 224 (0.03) | 0.004 |  | 208 (0.02) | 224 (0.03) | 0.001 |
| Fluoroquinolones (FQ) \| penicillins with ß-lactamase inhibitors (PEN BETA) \| standard deviation (SD) \| Charlson comorbidity index (CCI) \| cardiovascular diseases (CVD). | | | | | | | | |

eTable 1d. Study population characteristics before and after propensity score matching, cohort FQ vs 2^nd^/3^rd^ generation cephalosporins.

|  | | **Before Propensity Score Matching** | |  |  | **After Propensity Score Matching** | |  |
| --- | --- | --- | --- | --- | --- | --- | --- | --- |
|  | | **FQ**  ***(n = 2,121,502)*** | **CEPHA *(n = 2,716,997)*** | **Standardized difference** |  | **FQ *(n = 2,103,084)*** | **CEPHA *(n = 2,103,084)*** | **Standardized difference** |
| Age (mean (SD)) | | 56.51 (19.32) | 50.84 (19.61) | 0.291 |  | 56.40 (19.37) | 55.66 (18.99) | 0.038 |
| Male gender (%) | | 868,490 (40.94) | 1,210,290 (44.55) | 0.073 |  | 867,107 (41.23) | 850,480 (40.44) | 0.016 |
| CCI (%) | |  |  | 0.104 |  |  |  | 0.020 |
| 0 | | 1,295,906 (61.08) | 1,783,102 (65.63) |  |  | 1,280,386 (60.88) | 1,296,251 (61.64) |  |
| 1-2 | | 615,584 (29.02) | 724,936 (26.68) |  |  | 613,903 (29.19) | 608,140 (28.92) |  |
| 3-4 | | 162,410 (7.66) | 164,897 (6.07) |  |  | 161,437 (7.68) | 155,586 (7.40) |  |
| 5+ | | 47,602 (2.24) | 44,062 (1.62) |  |  | 47,358 (2.25) | 43,107 (2.05) |  |
| Drugs dispensed (%) | |  |  | 0.181 |  |  |  | 0.019 |
| 0 | | 330,966 (15.60) | 544,031 (20.02) |  |  | 330,960 (15.74) | 334,038 (15.88) |  |
| 1-3 | | 436,333 (20.57) | 657,808 (24.21) |  |  | 432,405 (20.56) | 442,326 (21.03) |  |
| 4-10 | | 518,373 (24.43) | 645,592 (23.76) |  |  | 514,910 (24.48) | 519,543 (24.70) |  |
| 11-20 | | 382,195 (18.02) | 405,973 (14.94) |  |  | 373,058 (17.74) | 368,959 (17.54) |  |
| 21+ | | 453,635 (21.38) | 463,593 (17.06) |  |  | 451,751 (21.48) | 438,218 (20.84) |  |
| Diabetes mellitus (%) | E10-14 | 410,669 (19.36) | 412,295 (15.18) | 0.111 |  | 408,740 (19.44) | 391,291 (18.61) | 0.021 |
| Cerebral vascular syndromes (%) | G45 | 19,816 (0.93) | 19,207 (0.71) | 0.025 |  | 19,749 (0.94) | 18,414 (0.88) | 0.007 |
|  | G46 | 1,345 (0.06) | 1,429 (0.05) | 0.004 |  | 1,341 (0.06) | 1,315 (0.06) | <0.001 |
| Hypertension (%) | I11 | 117,528 (5.54) | 115,982 (4.27) | 0.059 |  | 117,174 (5.57) | 111,375 (5.30) | 0.012 |
|  | I13 | 4,425 (0.21) | 4,098 (0.15) | 0.014 |  | 4,417 (0.21) | 3,983 (0.19) | 0.005 |
| Ischemic heart disease (%) | I20 | 38,748 (1.83) | 37,642 (1.39) | 0.035 |  | 38,502 (1.83) | 35,808 (1.70) | 0.010 |
|  | I22 | 472 (0.02) | 549 (0.02) | 0.001 |  | 471 (0.02) | 474 (0.02) | <0.001 |
|  | I24 | 4,879 (0.23) | 4,830 (0.18) | 0.012 |  | 4,871 (0.23) | 4,596 (0.22) | 0.003 |
|  | I25 | 247,471 (11.67) | 241,009 (8.87) | 0.092 |  | 245,960 (11.70) | 232,567 (11.06) | 0.020 |
| Valve diseases (%) | I34 | 70,978 (3.35) | 73,798 (2.72) | 0.037 |  | 70,802 (3.37) | 68,748 (3.27) | 0.005 |
|  | I35 | 57,298 (2.70) | 59,073 (2.17) | 0.034 |  | 57,107 (2.72) | 55,763 (2.65) | 0.004 |
|  | I36 | 7,498 (0.35) | 7,826 (0.29) | 0.012 |  | 7,477 (0.36) | 7,243 (0.34) | 0.002 |
|  | I37 | 2,440 (0.12) | 2,831 (0.10) | 0.003 |  | 2,432 (0.12) | 2,325 (0.11) | 0.002 |
| Cardiomyopathy (%) | I42 | 18,740 (0.88) | 21,415 (0.79) | 0.010 |  | 18,696 (0.89) | 18,497 (0.88) | 0.001 |
|  | I43 | 959 (0.05) | 1,029 (0.04) | 0.004 |  | 958 (0.05) | 924 (0.04) | 0.001 |
| Cardiac arrhythmia (%) | I44 | 33,728 (1.59) | 34,155 (1.26) | 0.028 |  | 33,633 (1.60) | 32,229 (1.53) | 0.005 |
|  | I45 | 21,861 (1.03) | 23,922 (0.88) | 0.015 |  | 21,823 (1.04) | 21,277 (1.01) | 0.003 |
|  | I46 | 481 (0.02) | 521 (0.02) | 0.002 |  | 479 (0.02) | 462 (0.02) | 0.001 |
|  | I47 | 24,512 (1.16) | 26,421 (0.97) | 0.018 |  | 24,229 (1.15) | 23,640 (1.12) | 0.003 |
|  | I48 | 105,150 (4.96) | 110,702 (4.07) | 0.042 |  | 104,688 (4.98) | 105,074 (5.00) | 0.001 |
|  | I49 | 129,689 (6.11) | 135,215 (4.98) | 0.050 |  | 129,409 (6.15) | 124,822 (5.94) | 0.009 |
| Heart failure (%) | I50 | 152,715 (7.20) | 154,077 (5.67) | 0.062 |  | 152,017 (7.23) | 146,590 (6.97) | 0.010 |
| Cerebrovascular diseases (%) | I61 | 3,139 (0.15) | 3,142 (0.12) | 0.009 |  | 3,124 (0.15) | 2,892 (0.14) | 0.003 |
|  | I62 | 1,034 (0.05) | 1,130 (0.04) | 0.003 |  | 1,032 (0.05) | 1,014 (0.05) | <0.001 |
|  | I63 | 21,214 (1.00) | 21,578 (0.79) | 0.022 |  | 21,147 (1.01) | 20,350 (0.97) | 0.004 |
|  | I64 | 20,752 (0.98) | 19,860 (0.73) | 0.027 |  | 20,711 (0.99) | 19,196 (0.91) | 0.007 |
|  | I65 | 54,031 (2.55) | 52,497 (1.93) | 0.042 |  | 53,852 (2.56) | 51,183 (2.43) | 0.008 |
|  | I66 | 2,550 (0.12) | 2,714 (0.10) | 0.006 |  | 2,541 (0.12) | 2,440 (0.12) | 0.001 |
|  | I67 | 71,670 (3.38) | 68,164 (2.51) | 0.051 |  | 71,409 (3.40) | 66,319 (3.15) | 0.014 |
|  | I68 | 215 (0.03) | 194 (0.03) | 0.002 |  | 215 (0.03) | 188 (0.03) | 0.002 |
| Diseases of the arteries, arterioles and capillaries (%) | I70 | 128,683 (6.07) | 123,836 (4.56) | 0.067 |  | 128,276 (6.10) | 120,663 (5.74) | 0.015 |
|  | I72 | 3,030 (0.14) | 3,215 (0.12) | 0.007 |  | 3,024 (0.14) | 2,950 (0.14) | 0.001 |
|  | I73 | 65,177 (3.07) | 65,538 (2.41) | 0.040 |  | 64,965 (3.09) | 62,845 (2.99) | 0.006 |
|  | I74 | 4,186 (0.20) | 4,345 (0.16) | 0.009 |  | 4,176 (0.20) | 4,045 (0.19) | 0.001 |
|  | I77 | 8,600 (0.41) | 8,863 (0.33) | 0.013 |  | 8,587 (0.41) | 8,282 (0.39) | 0.002 |
| Pulmonary edema (%) | J81 | 1,062 (0.05) | 1,120 (0.04) | 0.004 |  | 1,061 (0.05) | 1,039 (0.05) | <0.001 |
| Vascular diseases of the intestine (%) | K55 | 673 (0.03) | 668 (0.03) | 0.004 |  | 670 (0.03) | 615 (0.03) | 0.001 |
| Fluoroquinolones (FQ) \| 2nd/3rd generation cephalosporins (CEPHA) \| standard deviation (SD) \| Charlson comorbidity index (CCI) \| cardiovascular diseases (CVD). | | | | | | | | |

eTable 1e. Study population characteristics before and after propensity score matching, cohort FQ vs sulfonamide and trimethoprim combinations.

|  | | **Before Propensity Score Matching** | |  |  | **After Propensity Score Matching** | |  |
| --- | --- | --- | --- | --- | --- | --- | --- | --- |
|  | | **FQ**  ***(n = 2,121,502)*** | **COTRIM *(n = 528,618)*** | **Standardized difference** |  | **FQ  *(n = 528,613)*** | **COTRIM *(n = 528,613)*** | **Standardized difference** |
| Age (mean (SD)) | | 56.51 (19.32) | 55.25 (20.56) | 0.063 |  | 55.13 (20.47) | 55.25 (20.56) | 0.006 |
| Male gender (%) | | 868,490 (40.94) | 120,300 (22.76) | 0.398 |  | 120,308 (22.76) | 120,300 (22.76) |  |
| CCI (%) | |  |  | 0.082 |  |  |  | 0.005 |
| 0 | | 1,295,906 (61.08) | 343,719 (65.02) |  |  | 343,332 (64.95) | 343,719 (65.02) |  |
| 1-2 | | 615,584 (29.02) | 139,232 (26.34) |  |  | 139,857 (26.46) | 139,232 (26.34) |  |
| 3-4 | | 162,410 (7.66) | 35,323 (6.68) |  |  | 35,365 (6.69) | 35,323 (6.68) |  |
| 5+ | | 47,602 (2.24) | 10,339 (1.96) |  |  | 10,059 (1.90) | 10,339 (1.96) |  |
| Drugs dispensed (%) | |  |  | 0.043 |  |  |  | 0.001 |
| 0 | | 330,966 (15.60) | 84,711 (16.03) |  |  | 84,696 (16.02) | 84,711 (16.03) |  |
| 1-3 | | 436,333 (20.57) | 113,455 (21.46) |  |  | 113,645 (21.50) | 113,455 (21.46) |  |
| 4-10 | | 518,373 (24.43) | 132,887 (25.14) |  |  | 132,744 (25.11) | 132,887 (25.14) |  |
| 11-20 | | 382,195 (18.02) | 92,004 (17.41) |  |  | 91,868 (17.38) | 92,004 (17.41) |  |
| 21+ | | 453,635 (21.38) | 105,556 (19.97) |  |  | 105,660 (19.99) | 105,556 (19.97) |  |
| Diabetes mellitus (%) | E10-14 | 410,669 (19.36) | 94,163 (17.81) | 0.040 |  | 93,913 (17.77) | 94,163 (17.81) | 0.001 |
| Cerebral vascular syndromes (%) | G45 | 19,816 (0.93) | 4,675 (0.88) | 0.005 |  | 4,129 (0.78) | 4,675 (0.88) | 0.011 |
|  | G46 | 1,345 (0.06) | 396 (0.08) | 0.004 |  | 328 (0.06) | 396 (0.08) | 0.005 |
| Hypertension (%) | I11 | 117,528 (5.54) | 25,678 (4.86) | 0.031 |  | 24,734 (4.68) | 25,678 (4.86) | 0.008 |
|  | I13 | 4,425 (0.21) | 765 (0.15) | 0.015 |  | 775 (0.15) | 765 (0.15) | <0.001 |
| Ischemic heart disease (%) | I20 | 38,748 (1.83) | 8,296 (1.57) | 0.020 |  | 7,706 (1.46) | 8,296 (1.57) | 0.009 |
|  | I22 | 472 (0.02) | 85 (0.02) | 0.004 |  | 70 (0.01) | 85 (0.02) | 0.002 |
|  | I24 | 4,879 (0.23) | 986 (0.19) | 0.010 |  | 847 (0.16) | 986 (0.19) | 0.006 |
|  | I25 | 247,471 (11.67) | 50,308 (9.52) | 0.070 |  | 49,407 (9.35) | 50,308 (9.52) | 0.006 |
| Valve diseases (%) | I34 | 70,978 (3.35) | 16,755 (3.17) | 0.010 |  | 15,337 (2.90) | 16,755 (3.17) | 0.016 |
|  | I35 | 57,298 (2.70) | 12,948 (2.45) | 0.016 |  | 11,512 (2.18) | 12,948 (2.45) | 0.018 |
|  | I36 | 7,498 (0.35) | 1,704 (0.32) | 0.005 |  | 1,404 (0.27) | 1,704 (0.32) | 0.010 |
|  | I37 | 2,440 (0.12) | 564 (0.11) | 0.002 |  | 496 (0.09) | 564 (0.11) | 0.004 |
| Cardiomyopathy (%) | I42 | 18,740 (0.88) | 3,577 (0.68) | 0.023 |  | 3,139 (0.59) | 3,577 (0.68) | 0.010 |
|  | I43 | 959 (0.05) | 179 (0.03) | 0.006 |  | 152 (0.03) | 179 (0.03) | 0.003 |
| Cardiac arrhythmia (%) | I44 | 33,728 (1.59) | 7,494 (1.42) | 0.014 |  | 6,642 (1.26) | 7,494 (1.42) | 0.014 |
|  | I45 | 21,861 (1.03) | 4,606 (0.87) | 0.016 |  | 4,215 (0.80) | 4,606 (0.87) | 0.008 |
|  | I46 | 481 (0.02) | 97 (0.02) | 0.003 |  | 89 (0.02) | 97 (0.02) | 0.001 |
|  | I47 | 24,512 (1.16) | 6,112 (1.16) | <0.001 |  | 5,722 (1.08) | 6,112 (1.16) | 0.007 |
|  | I48 | 105,150 (4.96) | 23,299 (4.41) | 0.026 |  | 22,328 (4.22) | 23,299 (4.41) | 0.009 |
|  | I49 | 129,689 (6.11) | 30,403 (5.75) | 0.015 |  | 29,211 (5.53) | 30,403 (5.75) | 0.010 |
| Heart failure (%) | I50 | 152,715 (7.20) | 34,927 (6.61) | 0.023 |  | 34,001 (6.43) | 34,927 (6.61) | 0.007 |
| Cerebrovascular diseases (%) | I61 | 3,139 (0.15) | 819 (0.16) | 0.002 |  | 704 (0.13) | 819 (0.16) | 0.006 |
|  | I62 | 1,034 (0.05) | 275 (0.05) | 0.001 |  | 216 (0.04) | 275 (0.05) | 0.005 |
|  | I63 | 21,214 (1.00) | 5,076 (0.96) | 0.004 |  | 4,615 (0.87) | 5,076 (0.96) | 0.009 |
|  | I64 | 20,752 (0.98) | 4,800 (0.91) | 0.007 |  | 4,396 (0.83) | 4,800 (0.91) | 0.008 |
|  | I65 | 54,031 (2.55) | 11,795 (2.23) | 0.021 |  | 10,835 (2.05) | 11,795 (2.23) | 0.013 |
|  | I66 | 2,550 (0.12) | 589 (0.11) | 0.003 |  | 501 (0.10) | 589 (0.11) | 0.005 |
|  | I67 | 71,670 (3.38) | 16,863 (3.19) | 0.011 |  | 15,967 (3.02) | 16,863 (3.19) | 0.010 |
|  | I68 | 215 (0.03) | 52 (0.03) | 0.001 |  | 46 (0.03) | 52 (0.03) | 0.002 |
| Diseases of the arteries, arterioles and capillaries (%) | I70 | 128,683 (6.07) | 27,620 (5.23) | 0.036 |  | 26,885 (5.09) | 27,620 (5.23) | 0.006 |
|  | I72 | 3,030 (0.14) | 653 (0.12) | 0.005 |  | 611 (0.12) | 653 (0.12) | 0.002 |
|  | I73 | 65,177 (3.07) | 13,528 (2.56) | 0.031 |  | 13,406 (2.54) | 13,528 (2.56) | 0.001 |
|  | I74 | 4,186 (0.20) | 889 (0.17) | 0.007 |  | 766 (0.15) | 889 (0.17) | 0.006 |
|  | I77 | 8,600 (0.41) | 1,881 (0.36) | 0.008 |  | 1,706 (0.32) | 1,881 (0.36) | 0.006 |
| Pulmonary edema (%) | J81 | 1,062 (0.05) | 232 (0.04) | 0.003 |  | 194 (0.04) | 232 (0.04) | 0.004 |
| Vascular diseases of the intestine (%) | K55 | 673 (0.03) | 161 (0.03) | 0.001 |  | 129 (0.02) | 161 (0.03) | 0.004 |
| Fluoroquinolones (FQ) \| sulphonamide and trimethoprim combinations (COTRIM) \| standard deviation (SD) \| Charlson comorbidity index (CCI) \| cardiovascular diseases (CVD.) | | | | | | | | |

eTable 1f. Study population characteristics before and after propensity score matching, cohort FQ vs lincosamides.

|  | | **Before Propensity Score Matching** | |  |  | **After Propensity Score Matching** | |  |
| --- | --- | --- | --- | --- | --- | --- | --- | --- |
|  | | **FQ**  ***(n = 2,121,502)*** | **LINCO *(n = 1,166,520)*** | **Standardized difference** |  | **FQ *(n = 1,166,519)*** | **LINCO *(n = 1,166,519)*** | **Standardized difference** |
| Age (mean (SD)) | | 56.51 (19.32) | 49.43 (17.40) | 0.385 |  | 51.59 (18.40) | 49.43 (17.40) | 0.121 |
| Male gender (%) | | 868,490 (40.94) | 573,271 (49.14) | 0.166 |  | 651,215 (55.83) | 573,270 (49.14) | 0.134 |
| CCI (%) | |  |  | 0.263 |  |  |  | 0.134 |
| 0 | | 1,295,906 (61.08) | 848,420 (72.73) |  |  | 782,366 (67.07) | 848,419 (72.73) |  |
| 1-2 | | 615,584 (29.02) | 256,548 (21.99) |  |  | 294,815 (25.27) | 256,548 (21.99) |  |
| 3-4 | | 162,410 (7.66) | 48,635 (4.17) |  |  | 69,839 (5.99) | 48,635 (4.17) |  |
| 5+ | | 47,602 (2.24) | 12,917 (1.11) |  |  | 19,499 (1.67) | 12,917 (1.11) |  |
| Drugs dispensed (%) | |  |  | 0.350 |  |  |  | 0.165 |
| 0 | | 330,966 (15.60) | 292,621 (25.09) |  |  | 275,828 (23.65) | 292,620 (25.09) |  |
| 1-3 | | 436,333 (20.57) | 299,899 (25.71) |  |  | 232,840 (19.96) | 299,899 (25.71) |  |
| 4-10 | | 518,373 (24.43) | 273,813 (23.47) |  |  | 294,729 (25.27) | 273,813 (23.47) |  |
| 11-20 | | 382,195 (18.02) | 157,437 (13.50) |  |  | 194,948 (16.71) | 157,437 (13.50) |  |
| 21+ | | 453,635 (21.38) | 142,750 (12.24) |  |  | 168,174 (14.42) | 142,750 (12.24) |  |
| Diabetes mellitus (%) | E10-14 | 410,669 (19.36) | 147,412 (12.64) | 0.184 |  | 184,509 (15.82) | 147,412 (12.64) | 0.091 |
| Cerebral vascular syndromes (%) | G45 | 19,816 (0.93) | 6,377 (0.55) | 0.045 |  | 11,172 (0.96) | 6,377 (0.55) | 0.048 |
|  | G46 | 1,345 (0.06) | 477 (0.04) | 0.010 |  | 842 (0.07) | 477 (0.04) | 0.013 |
| Hypertension (%) | I11 | 117,528 (5.54) | 39,030 (3.35) | 0.107 |  | 64,369 (5.52) | 39,030 (3.35) | 0.106 |
|  | I13 | 4,425 (0.21) | 1,199 (0.10) | 0.027 |  | 2,214 (0.19) | 1,199 (0.10) | 0.023 |
| Ischemic heart disease (%) | I20 | 38,748 (1.83) | 13,140 (1.13) | 0.058 |  | 22,220 (1.91) | 13,140 (1.13) | 0.064 |
|  | I22 | 472 (0.02) | 190 (0.02) | 0.004 |  | 356 (0.03) | 190 (0.02) | 0.009 |
|  | I24 | 4,879 (0.23) | 1,548 (0.13) | 0.023 |  | 2,925 (0.25) | 1,548 (0.13) | 0.027 |
|  | I25 | 247,471 (11.67) | 78,716 (6.75) | 0.171 |  | 122,852 (10.53) | 78,716 (6.75) | 0.135 |
| Valve diseases (%) | I34 | 70,978 (3.35) | 26,332 (2.26) | 0.066 |  | 44,766 (3.84) | 26,332 (2.26) | 0.092 |
|  | I35 | 57,298 (2.70) | 20,881 (1.79) | 0.062 |  | 35,303 (3.03) | 20,881 (1.79) | 0.081 |
|  | I36 | 7,498 (0.35) | 2,948 (0.25) | 0.018 |  | 5,248 (0.45) | 2,947 (0.25) | 0.033 |
|  | I37 | 2,440 (0.12) | 1,107 (0.10) | 0.006 |  | 1,792 (0.15) | 1,106 (0.10) | 0.017 |
| Cardiomyopathy (%) | I42 | 18,740 (0.88) | 7,212 (0.62) | 0.031 |  | 12,959 (1.11) | 7,212 (0.62) | 0.053 |
|  | I43 | 959 (0.05) | 367 (0.03) | 0.007 |  | 666 (0.06) | 367 (0.03) | 0.012 |
| Cardiac arrhythmia (%) | I44 | 33,728 (1.59) | 11,508 (0.99) | 0.054 |  | 20,454 (1.75) | 11,508 (0.99) | 0.066 |
|  | I45 | 21,861 (1.03) | 8,907 (0.76) | 0.028 |  | 15,063 (1.29) | 8,907 (0.76) | 0.052 |
|  | I46 | 481 (0.02) | 157 (0.01) | 0.007 |  | 311 (0.03) | 157 (0.01) | 0.009 |
|  | I47 | 24,512 (1.16) | 10,260 (0.88) | 0.027 |  | 15,463 (1.33) | 10,260 (0.88) | 0.043 |
|  | I48 | 105,150 (4.96) | 31,827 (2.73) | 0.116 |  | 52,499 (4.50) | 31,827 (2.73) | 0.095 |
|  | I49 | 129,689 (6.11) | 47,227 (4.05) | 0.094 |  | 74,602 (6.40) | 47,227 (4.05) | 0.106 |
| Heart failure (%) | I50 | 152,715 (7.20) | 41,817 (3.59) | 0.161 |  | 67,666 (5.80) | 41,817 (3.59) | 0.105 |
| Cerebrovascular diseases (%) | I61 | 3,139 (0.15) | 986 (0.09) | 0.019 |  | 1,778 (0.15) | 986 (0.09) | 0.020 |
|  | I62 | 1,034 (0.05) | 339 (0.03) | 0.010 |  | 625 (0.05) | 339 (0.03) | 0.012 |
|  | I63 | 21,214 (1.00) | 6,649 (0.57) | 0.049 |  | 11,405 (0.98) | 6,649 (0.57) | 0.047 |
|  | I64 | 20,752 (0.98) | 5,987 (0.51) | 0.054 |  | 10,498 (0.90) | 5,987 (0.51) | 0.046 |
|  | I65 | 54,031 (2.55) | 18,789 (1.61) | 0.066 |  | 32,698 (2.80) | 18,789 (1.61) | 0.081 |
|  | I66 | 2,550 (0.12) | 983 (0.08) | 0.011 |  | 1,741 (0.15) | 983 (0.08) | 0.019 |
|  | I67 | 71,670 (3.38) | 21,299 (1.83) | 0.098 |  | 37,553 (3.22) | 21,299 (1.83) | 0.089 |
|  | I68 | 215 (0.03) | 62 (0.03) | 0.004 |  | 105 (0.03) | 62 (0.03) | 0.002 |
| Diseases of the arteries, arterioles and capillaries (%) | I70 | 128,683 (6.07) | 43,619 (3.74) | 0.108 |  | 70,735 (6.06) | 43,619 (3.74) | 0.108 |
|  | I72 | 3,030 (0.14) | 1,187 (0.10) | 0.012 |  | 2,186 (0.19) | 1,187 (0.10) | 0.023 |
|  | I73 | 65,177 (3.07) | 22,172 (1.90) | 0.075 |  | 39,177 (3.36) | 22,172 (1.90) | 0.091 |
|  | I74 | 4,186 (0.20) | 1,531 (0.13) | 0.016 |  | 2,834 (0.24) | 1,531 (0.13) | 0.026 |
|  | I77 | 8,600 (0.41) | 3,166 (0.27) | 0.023 |  | 5,631 (0.48) | 3,166 (0.27) | 0.034 |
| Pulmonary edema (%) | J81 | 1,062 (0.05) | 225 (0.02) | 0.017 |  | 464 (0.04) | 225 (0.02) | 0.012 |
| Vascular diseases of the intestine (%) | K55 | 673 (0.03) | 217 (0.02) | 0.008 |  | 415 (0.04) | 217 (0.02) | 0.010 |
| Fluoroquinolones (FQ) \| lincosamides (LINCO) \| standard deviation (SD) \| Charlson comorbidity index (CCI) \| cardiovascular diseases (CVD). | | | | | | | | |

eTable 2. Results from Cox regression, frailty model, FQ vs. macrolides.

|  | **Aortic aneurysm / dissection** | | | |
| --- | --- | --- | --- | --- |
|  | **aHR** | | **[95% CI]** | |
| FQ episode (ref. AC) | 1.520 | | [1.329;1.739] | |
| Age in years | 1.065 | | [1.060;1.071] | |
| Males (ref. females) | 3.823 | | [3.314;4.409] | |
| CCI (ref. 0) |  | |  | |
| 1-2 | 1.100 | | [0.948;1.275] | |
| 3-4 | 1.170 | | [0.958;1.429] | |
| 5+ | 0.872 | | [0.612;1.242] | |
| CVD | 1.513 | | [1.293;1.772] | |
| Diabetes mellitus | 0.719 | | [0.718;0.965] | |
| *Variance of random effect* | | *1.409* |  |  |
| Adjusted hazard ratio (aHR) \| 95% confidence interval (CI) [lower bound; bound] \| reference (ref.) \| Fluoroquinolone (FQ) \| active comparator (AC) \| Charlson comorbidity index (CCI) \| cardiovascular disease (CVD). | | | | |

eTable 3. Results from Cox regression, frailty model, FQ vs. other active comparators.

|  | **Aortic aneurysm / dissection** | | *Variance of random effect* |
| --- | --- | --- | --- |
|  |  | |  |
|  | **aHR** | **[95% CI]** |  |
| FQ vs. tetracyclines | 1.859 | [1.542;2.241] | *0.0004* |
| FQ vs. penicillins with extended spectrum | 1.452 | [1.276;1.652] | *1.3970* |
| FQ vs. penicillins and beta-lactamase inhibitors | 0.898 | [0.764;1.056] | *0.0004* |
| FQ vs. 2nd and 3rd generation cephalosporins | 1.229 | [1.104;1.367] | *0.7250* |
| FQ vs. sulfonamide and trimethoprim | 0.909 | [0.717;1.153] | *0.0004* |
| FQ vs. lincosamides | 1.737 | [1.430;2.110] | *0.0004* |
| Adjusted hazard ratio (aHR) \| 95% confidence interval (CI) [lower bound; upper bound] \| reference (ref.) \| Fluoroquinolone (FQ) \| active comparator (AC) \| regressions were adjusted for age, gender, Charlson comorbidity index, cardiovascular diseases, and diabetes mellitus. | | |  |
